# Immunohistochemical assessment and clinical, histopathologic, and molecular correlates of membranous somatostatin type-2A receptor expression in high-risk pediatric central nervous system tumors

**DOI:** 10.3389/fonc.2022.996489

**Published:** 2022-11-17

**Authors:** Margot A. Lazow, Christine Fuller, Andrew T. Trout, Joseph R. Stanek, Jaime Reuss, Brian K. Turpin, Sara Szabo, Ralph Salloum

**Affiliations:** ^1^ Pediatric Neuro-Oncology Program, Nationwide Children’s Hospital, Columbus, OH, United States; ^2^ Department of Pediatrics, The Ohio State University College of Medicine, Columbus, OH, United States; ^3^ Cancer and Blood Diseases Institute, Cincinnati Children’s Hospital Medical Center, Cincinnati, OH, United States; ^4^ Department of Pathology, Upstate Medical University, Syracuse, NY, United States; ^5^ Department of Radiology and Medical Imaging, Cincinnati Children’s Hospital Medical Center, Cincinnati, OH, United States; ^6^ Department of Radiology, University of Cincinnati College of Medicine, Cincinnati, OH, United States; ^7^ Department of Pediatrics, University of Cincinnati College of Medicine, Cincinnati, OH, United States; ^8^ Department of Pathology, Cincinnati Children’s Hospital Medical Center, Cincinnati, OH, United States

**Keywords:** somatostatin receptor, SST2A, immunohistochemistry, pediatric CNS tumors, embryonal tumors, medulloblastoma, somatostatin receptor-targeted therapy, DOTATATE

## Abstract

**Introduction:**

^177^Lu-DOTATATE, a radionuclide therapy that binds somatostatin type-2A receptors (SST2A), has demonstrated efficacy in neuroendocrine tumors and evidence of central nervous system (CNS) penetration, supporting potential expansion within pediatric neuro-oncology. Understanding the prevalence of SST2A expression across pediatric CNS tumors is essential to identify patients who may benefit from somatostatin receptor-targeted therapy and to further elucidate the oncogenic role of SST2A.

**Methods:**

SST2A immunohistochemistry (IHC) was performed on tumor specimens and interpreted by an experienced pathologist (blinded), utilizing semi-quantitative scoring of membranous expression within viable tumor. Immunoreactive cell percentage was visually scored as 0 (none), 1 (<10%), 2 (10-50%), 3 (51-80%), or 4 (>80%). Staining intensity was scored as 0 (none), 1 (weak), 2 (moderate), or 3 (strong). Combined scores for each specimen were calculated by multiplying percent immunoreactivity and staining intensity values (Range: 0-12).

**Results:**

A total of 120 tumor samples from 114 patients were analyzed. Significant differences in SST2A IHC scores were observed across histopathologic diagnoses, with consistently high scores in medulloblastoma (mean ± SD: 7.5 ± 3.6 [n=38]) and meningioma (5.7 ± 3.4 [n=15]), compared to minimal or absent expression in ATRT (0.3 ± 0.6 [n=3]), ETMR (1.0 ± 0 [n=3]), ependymoma (grades I-III; 0.2 ± 0.7 [n=27]), and high-grade glioma (grades III-IV; 0.4 ± 0.7 [n=23]). Pineoblastoma (3.8 ± 1.5 [n=4]) and other embryonal tumors (2.0 ± 4.0 [n=7]) exhibited intermediate, variable expression. Among medulloblastomas, SST2A IHC scores were higher in non-SHH (8.5 ± 3.1) than SHH (5.0 ± 3.3) molecular subgroups (p=0.033). In a subset of paired primary and recurrent specimens from four patients, SST2A IHC scores remained largely unchanged.

**Discussion:**

High membranous SST2A expression was demonstrated in medulloblastoma, meningioma, and some rarer embryonal tumors with potential diagnostic, biologic, and therapeutic implications. Somatostatin receptor-targeted therapy such as ^177^Lu-DOTATATE deserves further investigation in these highly SST2A-expressing pediatric CNS tumors.

## Introduction

High-grade central nervous system (CNS) tumors remain a leading cause of cancer-related death in children and adolescents (1). While cure can sometimes be achieved with conventional chemotherapy, surgery, and/or radiation, the prognosis for patients with recurrent or progressive disease despite these treatments is dismal (2–6). There is therefore a critical need to develop new, effective therapies for pediatric patients with refractory CNS tumors. Somatostatin receptors regulate cell growth through complex downstream modulation of both proliferation (i.e., mitogen-activated protein kinase, protein tyrosine phosphatase) and apoptosis signaling pathways, and thus represent a potential therapeutic target (7–9). Lutetium (^177^Lu)-DOTATATE, a radionuclide therapy which binds type-2A somatostatin receptors (SST2A) and delivers local radiation *via* beta particle emission, has gained FDA approval for the treatment of adult patients with gastroenteropancreatic neuroendocrine tumors (10, 11), a disease characterized by consistent SST2A expression (8). There is emerging evidence that certain pediatric CNS tumors express SST2A, with corresponding uptake on somatostatin-receptor radiolabeled nuclear imaging (12–39). SST2A expression has been described in medulloblastoma (13, 26–31), other embryonal tumors (17, 28, 31), meningiomas (25, 32, 33), high-grade gliomas (17, 27, 34–38), and ependymomas (17, 39), though with variable frequencies and lower levels in the latter two histologic diagnoses. Case reports/series have demonstrated treatment response (disease stabilization or regression) to somatostatin receptor-targeted therapy in children and young adults with relapsed medulloblastoma, high-grade glioma, meningioma, and brain metastases of neuroendocrine tumors (19, 22–25, 40–46), suggesting sufficient CNS penetration to achieve therapeutic benefit.

Understanding the prevalence, heterogeneity, and key correlates of SST2A expression across pediatric high-grade CNS tumors is essential to determine which patients are most likely to respond and to further elucidate the oncogenic role of somatostatin receptor pathways within these aggressive diseases. Although aforementioned reports of SST2A expression in pediatric CNS tumors support investigation of somatostatin receptor-targeted therapy (13, 17, 27–38), findings were limited by small sample sizes, varied measures of receptor levels (including SST2A mRNA, an imperfect surrogate for functional protein expression) (47, 48), and inconsistent definitions of SST2A positivity by immunohistochemistry (IHC). Several prior studies evaluating SST2A expression by IHC in CNS tumors used polyclonal anti-SST2A antibodies, which may yield less specific results due to cross-reactivity with other antigens (49, 50). Moreover, associations between SST2A expression and tumor stage, histologic grade, presence of prognostically-significant genetic alterations, response to ^177^Lu-DOTATATE, and/or survival have been established in neuroendocrine tumors, neuroblastoma, and adult anaplastic oligodendrogliomas (36, 37, 48, 51–55), but corresponding data are lacking in pediatric CNS tumors.

Identifying patients with high-risk CNS tumors who may benefit from somatostatin receptor-targeted therapy demands rigorous assessment of membranous (i.e., targetable) SST2A protein expression *via* a validated, functionally-relevant SST2A IHC scoring system. Within a large, representative cohort of pediatric high-grade and/or difficult-to-treat CNS tumors, we applied SST2A IHC scoring methodology adapted from neuroendocrine tumors, which demonstrated correlation with somatostatin autoradiography quantification *in vitro* as well as uptake on somatostatin receptor nuclear imaging and response to somatostatin analog therapy *in vivo* (12, 49, 51, 56–58). Additionally, SST2A IHC was performed with a newer monoclonal anti-SST2A antibody (UMB-1), which offers improved sensitivity and specificity compared to earlier polyclonal antibodies (48–50). Utilizing these tools and scoring approach, we evaluated the prevalence of membranous SST2A expression and potential clinical, histopathologic, and molecular correlates across high-risk pediatric CNS tumors.

## Materials and methods

### Clinical cohort

This retrospective study was performed at Cincinnati Children’s Hospital Medical Center (CCHMC) and included pediatric, adolescent, and young adult patients enrolled in the CCHMC institutional review board-approved tumor tissue repository. The patient cohort was selected based on availability of adequate tumor specimens from diagnosis and/or recurrence, with a confirmed histologic diagnosis of CNS embryonal tumor, high-grade glial neoplasm, ependymoma (any grade), and meningioma (any grade) by pathology review. This histopathologic distribution was chosen to ensure inclusion of diagnoses with previously reported evidence suggesting SST2A expression as well as tumors with high histologic grade and/or limited therapeutic options at recurrence. The following clinical data were abstracted from patients’ electronic health records and subsequently de-identified: age, sex, presence of metastases, molecular profiling results if applicable, treatment details, event-free survival (defined as time from diagnosis to disease progression, recurrence, secondary malignancy, death, or censoring), and overall survival (defined as time from diagnosis to death or censoring). Patients without an event at last known follow-up were considered censored. All tumor samples and clinical data were collected after informed consent was provided by patients or legal guardians.

### Tissue and SST2A IHC preparation

Tumor samples had been preserved as formalin fixed paraffin embedded (FFPE) tissue. To ensure adequate tumor content and viability, hematoxylin and eosin (H&E) slides were first reviewed from the same FFPE block that SST2A IHC was to be performed. Four micron-thick sections were subjected to SST2A IHC preparation following the College of American Pathologists (CAP)/Clinical Laboratory Improvement Amendments (CLIA)-validated clinical assay utilized at CCHMC. Heat-Induced Epitope Retrieval was performed with Ethylenediaminetetraacetic acid and samples were stained, *via* an automated Ventana Ultra IHC stainer at a dilution of 1:200 using the monoclonal anti-SST2A antibody UMB-1 (a rabbit monoclonal antibody targeting the C-terminus of the SST2A protein [Abcam catalog# 134152]), as introduced above (49, 50). Samples were then processed using a secondary antibody and 3,3’-Diaminobenzidine chromogens (Roche: ultraView Universal DAB Detection Kit, catalog# 760-500) for signal visualization.

### SST2A IHC scoring and interpretation

**Figure 1:**
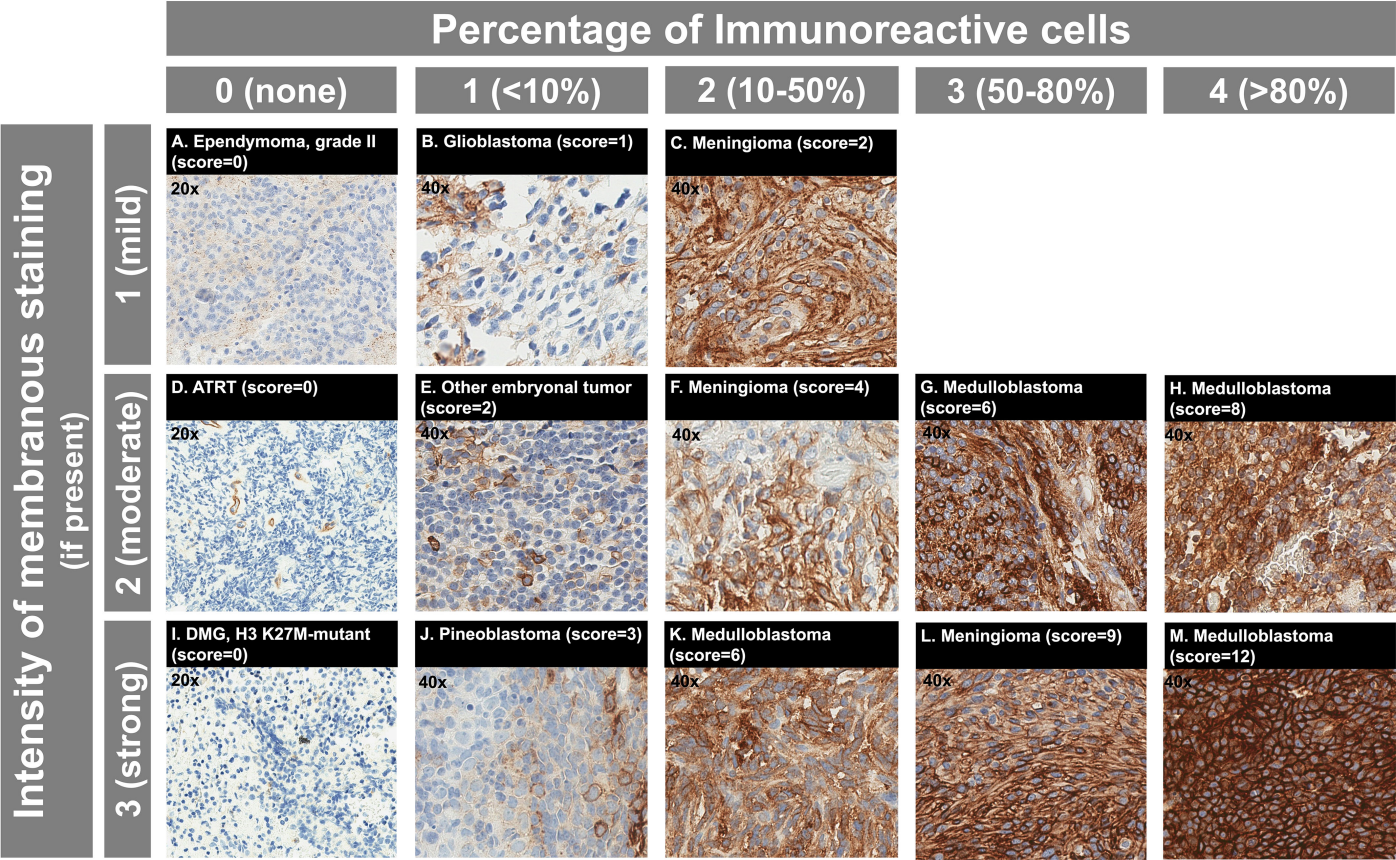
SST2A IHC scoring system with example cases from the analyzed pediatric CNS tumor cohort. **(A–M)** Immunoreactive cell percentage is illustrated horizontally, with scores ranging from 0 (none) to 4 (>80%) shown. Staining intensity is illustrated vertically, with scores ranging from 1 (mild) to 3 (strong) shown for tumors with membranous expression present. Total SST2A IHC score (calculated from multiplying immunoreactive cell percentage and staining intensity scores) is noted in parentheses for each example, with the respective histopathologic diagnosis specified. Note that all three cases in the first column **(A**, **D**, **I)** had entirely absent membranous SST2A expression within viable tumor and received total SST2A IHC scores of 0; images for these cases are shown at 20x magnification. Endothelial staining serves as a positive internal control **(D)**. For all other cases, images are shown at 40x magnification.

SST2A IHC was interpreted by an experienced pathologist (SS) together with a pediatric neuro-oncologist (MAL) in all cases, both blinded to clinical data. A semi-quantitative scoring system was utilized, which incorporated the following SST2A IHC staining profile characteristics: presence and completeness of membranous (versus cytoplasmic) staining, percent of immunoreactive tumor cells, and staining intensity (12, 49, 51, 56–58). Specifically, immunoreactive tumor cell percentage was visually scored as 0 (none), 1 (<10%), 2 (10-50%), 3 (51-80%), or 4 (>80%). Staining intensity was scored as 0 (none), 1 (weak), 2 (moderate), or 3 (strong). Combined scores for each specimen were calculated by multiplying percent immunoreactivity and staining intensity values (possible range: 0-12; **Figure 1**), as has been implemented in SST2A immunoreactivity assessments of neuroendocrine tumors (51), pituitary adenomas (58), and adult high-grade gliomas (37). Scores of 0-1 were considered negative; scores ≥2 were considered positive. Only membranous staining within viable tumor was considered for scoring purposes. However, non-membranous staining and/or staining in non-tumor cells were recorded for descriptive purposes (and the latter often provided internal negative or positive [e.g., endothelial] controls). Unevaluable areas of hemorrhagic or ischemic tumor were excluded from scoring. Staining distribution, heterogeneity within individual tumor specimens, morphologic patterns, and other relevant histopathologic features were also assessed. Additionally, to evaluate inter-rater reliability of this SST2A IHC scoring system within this pediatric CNS tumor cohort, a second pathologist (CF, blinded to the first pathologist’s scores) reviewed digitally uploaded SST2A IHC slides for a subset of 50 tumors, applying the same scoring rules. This sample was intentionally selected to include the range of histopathologic diagnoses from the entire cohort as well as a variety of specimens with high, intermediate, and minimal to absent membranous SST2A expression, as interpreted by the first pathologist. Cases with discordant impressions were subsequently re-examined by both reviewers together, with further collective inspection and discussion to reach consensus final score.

### Statistical analysis

Continuous and categorical variables are described by mean ( ± standard deviation [SD]) or median (range) and frequency (percent), respectively. T-tests and one-way ANOVA or Wilcoxon rank-sum and Kruskal Wallis tests were used for comparisons of mean SST2A IHC score based on specific clinical, histopathologic, and molecular features. Pearson’s correlation was used to evaluate associations between patient age and SST2A IHC within the medulloblastoma cohort. To assess potential associations between SST2A expression and outcome (event-free survival and overall survival, as defined above), univariate and multivariable Cox proportional hazards regression analyses were performed, with SST2A IHC score and histologic diagnosis as covariates; hazard ratios (HR) and corresponding 95% confidence intervals (CI) were reported. Survival outcomes were summarized using the Kaplan-Meier method and log-rank analyses were used to compare survival between patients divided into three SST2A IHC score categories (0-1 [negative], 2-5, 6-12). The weighted kappa statistic and Spearman’s correlation were used to evaluate measures of inter-reviewer reliability between pathologists, All p-values were two-sided and those less than 0.05 were considered statistically significant. Statistical analyses were completed in SAS software, version 9.4 (SAS Institute, Cary, NC) or base R statistical software (R Foundation for Statistical Computing, Vienna, Austria) with the “survival” and “survminer” packages.

## Results

### Cohort characteristics

A total of 120 tumor samples from 114 patients were included in the analysis. Demographic and clinical characteristics are summarized for the entire cohort and by histopathologic diagnosis in **Table 1**. Median age at diagnosis was 7.1 years (range: 0.1-29.3 years), and 39% of the patients were female. Metastatic disease was identified in 13% of patients at initial diagnosis, and 25% experienced subsequent recurrence or progression. Eighty-nine percent were alive at last follow-up (median 7.7 years from diagnosis).

**Table 1:** Cohort Characteristics.

	Number (%) of patients	Age at diagnosis (median [range] in years)	Gender distribution (n [%] female)	Metastatic disease at diagnosis (n [%])	Subsequent recurrence or progression (n [%])	Number (%) Alive at Last Follow-up	Time to last follow-up (median [range] in years)
**Entire cohort**	114	7.1 (0.1 – 29.3)	44 (39%)	15 (13%)	28 (25%)	102 (89%)	7.7 (0.2 – 30.3)
*By Histopathologic diagnosis:*							
**Meningioma**	13 (11%)	14.3 (6.0 – 29.3)	7 (54%)	0 (0%)	4 (31%)	13 (100%)	11.2 (0.8 – 25.5)
**Medulloblastoma**	38 (33%)	6.5 (0.9 – 17.6)	6 (16%)	8 (21%)	3 (8%)	37 (97%)*	9.0 (0.2 – 26.7)
**Pineoblastoma**	4 (4%)	6.8 (0.8 – 21.9)	3 (75%)	2 (50%)	0 (0%)	4 (100%)	2.3 (1.5 – 15.3)
**ATRT**	3 (3%)	1.3 (0.8 – 4.9)	2 (67%)	0 (0%)	0 (0%)	2 (67%)^#^	11.3 (1.3 – 13.8)
**ETMR**	3 (3%)	2.6 (2.2 – 3.7)	2 (67%)	1 (33%)	1 (33%)	2 (67%)	0.3 (0.3 – 6.9)
**Other embryonal tumors** ^a^	7 (6%)	2.1 (1.2 – 15.7)	2 (29%)	3 (43%)	2 (29%)	5 (71)	10.4 (1.8 – 22.1)
**Ependymoma**	25 (22%)	7.0 (1.0 – 18.1)	13 (52%)	0 (0%)	5 (20%)	25 (100%)	8.3 (0.7 – 30.3)
**High-grade glioma**	21 (18%)	10.3 (0.1 – 27.8)	9 (43%)	1 (5%)	13 (62%)	14 (67%)	4.4 (0.3 – 22.8)

Demographic and clinical features of patients included in the analysis, summarized overall and within histopathologic diagnosis subgroups.

a The subgroup designated “other embryonal tumors” includes six patients with tumors formerly classified as CNS primitive neuro-ectodermal tumor (PNET) and one patient more recently diagnosed with embryonal tumor, not otherwise specified (NOS).

*^,#^ Cause of death was disease recurrence or progression for deceased patients in the cohort except one patient with medulloblastoma (sequalae of treatment-related co-morbidities) and one patient with ATRT (complications of brainstem necrosis).

### Assessment of membranous SST2A expression across and within histopathologic diagnoses

#### Comparison across histologic subgroups:

**Figure 2:**
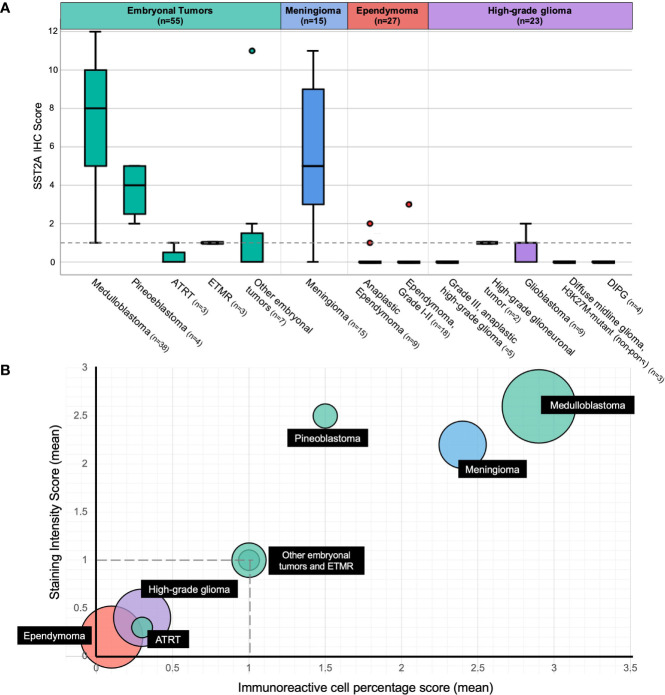
Membranous SST2A expression by histopathology. **(A)** Boxplot of total SST2A IHC score (y-axis) for pediatric CNS tumors of different histopathologic diagnoses (x-axis). The dashed gray line (corresponding to a total SST2A IHC score of 1) distinguishes between negative (0-1) and positive (≥2) SST2A IHC scores. **(B)** Bubble plot of mean SST2A IHC subscores (staining intensity score on y-axis, immunoreactivity score on x-axis) for different histopathologic diagnoses (indicated in black boxes), with circle size proportional to the number of patients analyzed within each respective histopathology group. The dashed horizontal and vertical gray lines (corresponding to staining intensity and immunoreactivity scores of 1, respectively) illustrate the subscore thresholds below which total SST2A IHC scores would be considered negative (0-1).

The distribution of membranous SST2A expression by histopathology is illustrated in **Figure 2** and summarized in **Table 2**, with significant differences in SST2A IHC scores between histopathologic diagnosis groups (p<0.001). Higher total SST2A IHC scores were observed among embryonal tumors (mean ± SD: 5.8 ± 4.2), albeit with variation described below, and meningioma (5.7 ± 3.4), compared to ependymomas (0.7 ± 0.1) and high-grade gliomas (0.4 ± 0.7), both with minimal or absent expression (**Figure 2**).

**Table 2:** Summary of SST2A IHC immunoreactive cell percentage, staining intensity, and combined scores based on histopathologic diagnosis.

	Immunoreactive cell percentage score (Range: 0-4)	Staining intensity score (Range: 0-3)	Combined SST2A IHC score (Range: 0-12)
**Entire cohort (n=120)**	1.4 (± 1.5)	1.4 (± 1.2)	3.5 (± 4.1)
**Meningioma (n=15)**	2.4 (± 1.2)	2.2 (± 0.9)	5.7 (± 3.4)
**Embryonal tumors (n=55)**	2.3 (± 1.4)	2.1 (± 1.0)	5.8 (± 4.2)
• **Medulloblastoma (n=38)**	2.9 (± 1.2)	2.6 (± 0.6)	7.5 (± 3.5)
• **Pineoblastoma (n=4)**	1.5 (± 0.6)	2.5 (± 0.4)	3.8 (± 1.5)
• **ATRT (n=3)**	0.3 (± 0.6)	0.3 (± 0.6)	0.3 (± 0.6)
• **ETMR (n=3)**	1.0 (± 0.0)	1.0 (± 0.0)	1.0 (± 0.0)
• **Other embryonal tumors (n=7)** ^a^	0.8 (± 1.3)	0.9 (± 1.2)	2.0 (± 4.0)
**Ependymoma (n=27)**	0.1 (± 0.3)	0.2 (± 0.7)	0.2 (± 0.7)
• **Ependymoma, grade I-II (n=18)**	0.1 (± 0.2)	0.2 (± 0.7)	0.3 (± 0.8)
• **Anaplastic ependymoma (n=9)**	0.2 (± 0.4)	0.3 (± 0.7)	0.1 (± 0.3)
**High-grade glioma (n=23)**	0.3 (± 0.5)	0.4 (± 0.7)	0.4 (± 0.7)
• Grade III, anaplastic glioma (n=5)*	0.0 (± 0.0)	0.0 (± 0.0)	0.0 (± 0.0)
• **High-grade glioneuronal tumor (n=2)**	1.0 (± 0.0)	1.0 (± 0.0)	1.0 (± 0.0)
• **Glioblastoma (n=9)**	0.7 (± 0.5)	0.9 (± 0.8)	0.9 (± 0.8)
• **Diffuse midline glioma, H3K27M-mutant (non-pontine) (n=3)**	0.0 (± 0.0)	0.0 (± 0.0)	0.0 (± 0.0)
• **DIPG (n=4)^#^**	0.0 (± 0.0)	0.0 (± 0.0)	0.0 (± 0.0)

Scores are shown as mean (± SD) for the entire cohort and within respective histopathologic diagnosis subgroups.

a The subgroup designated “other embryonal tumors” includes six patients with tumors formerly classified as CNS primitive neuro-ectodermal tumor (PNET) and one patient more recently diagnosed with embryonal tumor, not otherwise specified (NOS).

* Including tumors classified as anaplastic astrocytoma (n=3), anaplastic pleomorphic xanthoastrocytoma (n=1), and anaplastic oligodendroglioma (n=1).

^#^ Including tumors diagnosed as DIPG on the basis of classic radiographic and clinical features, histologically consistent with diffuse midline glioma, H3K27M-mutant (n=3) and anaplastic astrocytoma (n=1) [all with tissue available from biopsy].

### Embryonal tumors

#### Medulloblastoma

The 38 medulloblastoma samples collectively comprised the tumors with the highest SST2A expression in our cohort (**Figure 2**). Positive SST2A IHC scores (≥2) were reported in 35 (92%) medulloblastoma tumors (**Figures 1G, H, K, M**), with all 35 demonstrating moderate-strong staining intensity and most (22 [63%]) exhibiting >50% tumor cell immunoreactivity.

**Figure 3:**
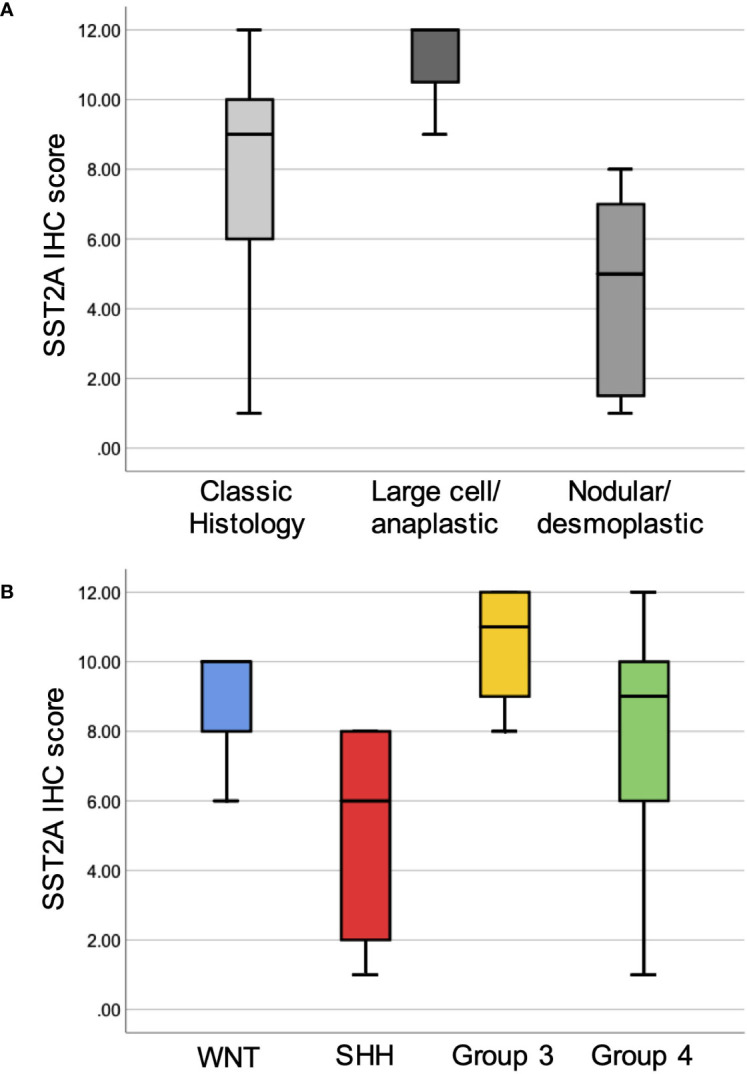
Membranous SST2A expression across histopathologic and molecular subgroups of medulloblastoma. Boxplots illustrate total SST2A IHC score by histologic **(A)** and molecular **(B)** classification.

There were significant differences in membranous SST2A expression between medulloblastoma histopathologic subgroups, with the highest SST2A IHC scores observed in large cell/anaplastic tumors (11 ± 1.7 [n=3]), followed by classic histology (7.9 ± 3.3 [n=17]], and lowest, yet often still positive, SST2A IHC scores in nodular/desmoplastic tumors (4.4 ± 3.1 [n=7]; p=0.012 for comparison across 3 groups; **Figure 3A**).

When comparing the 24 medulloblastoma specimens with available methylation results enabling molecular classification (**Figure 3B**), there was a trend toward differences in mean SST2A IHC score across the four molecular subgroups (group 3: 10.5 ± 1.9: [n=4] > WNT: 8.7 ± 2.3 [n=3] > group 4: 7.7 ± 3.5 [n=12] > Sonic Hedgehog (SHH): 5.0 ± 3.3 [n=5]; p=0.096), and significantly higher scores in non-SHH versus SHH tumors (8.5 ± 3.1 vs. 5.0 ± 3.3; p=0.033). Correspondingly, three of the four tumors with the lowest SST2A IHC scores (1–2) within the medulloblastoma cohort were classified as nodular/desmoplastic histologically, with two confirmed as SHH-activated by methylation testing (not performed in third).

An inverse correlation was observed between SST2A IHC score and patient age at diagnosis when analyzed across all 38 medulloblastoma cases (R=-0.32, p=0.048). There were no significant differences in membranous SST2A expression by sex (female: 9.5 ± 2.6 [n=6], male: 7.0 ± 3.6 [n=32]; p=0.13), presence of metastatic disease at diagnosis (metastatic: 6.9 ± 3.0 [n=8], localized: 7.6 ± 3.7 [n=30]; p=0.58), or likelihood of relapse (among patients followed ≥2 years from diagnosis: relapse: 9.0 ± 1.0 [n=3]; no relapse: 7.2 ± 3.8 [n=29]; p=0.45). One recurrent, post-treatment specimen was analyzed, with a SST2A IHC score of 10 (corresponding diagnostic sample was not available).

**Figure 4:**
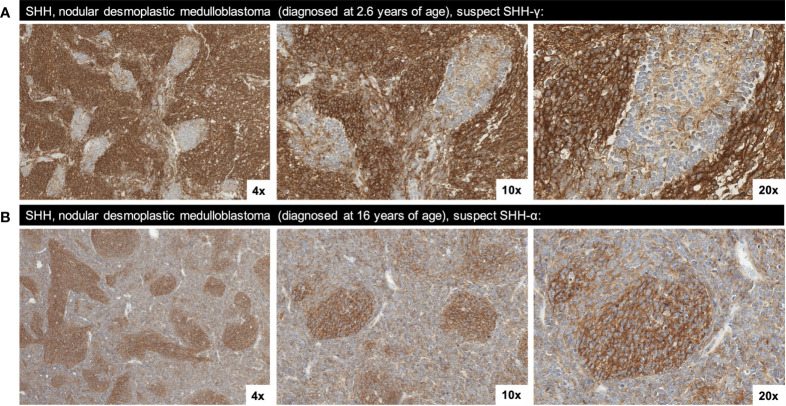
Two morphological patterns of SST2A staining observed in SHH, nodular/desmoplastic medulloblastoma. **(A)** SST2A IHC of SHH medulloblastoma (in a patient diagnosed at 2.6 years of age) demonstrating an inverse correlation between membranous SST2A expression and tumor cell maturation; the more immature cells exhibit strong, complete, circumferential SST2A IHC positivity, whereas the more mature cells comprising the nodules lack membranous expression (yet have cytoplasmic granularity). **(B)** SST2A IHC of SHH medulloblastoma (in a patient diagnosed at 16 years of age), with more primitive cells lacking membranous SST2A expression while the nodules’ differentiating cells exhibit SST2A positivity (though not consistently membranous). Images in both cases are shown at 4x, 10x, and 20x magnification.

Morphological patterns of SST2A expression were identified across medulloblastoma tumors and specifically within the nodular/desmoplastic subset, which enabled comparison of IHC positivity along the intratumoral spectrum of cell differentiation. In several cases, including two infant SHH tumors with higher SST2A IHC scores, SST2A expression inversely correlated with tumor cell maturation, with more immature cells demonstrating strong, complete, circumferential SST2A IHC positivity, compared with absent membranous expression in the more mature cells comprising the tumor nodules (**Figure 4A**). Conversely, in two other nodular/desmoplastic histology cases, including one adolescent SHH tumor with a low SST2A IHC score, the more primitive cells lacked SST2A positivity, whereas expression was observed within the nodules’ differentiating cells (**Figure 4B**).

#### Pineoblastoma

**Figure 5:**
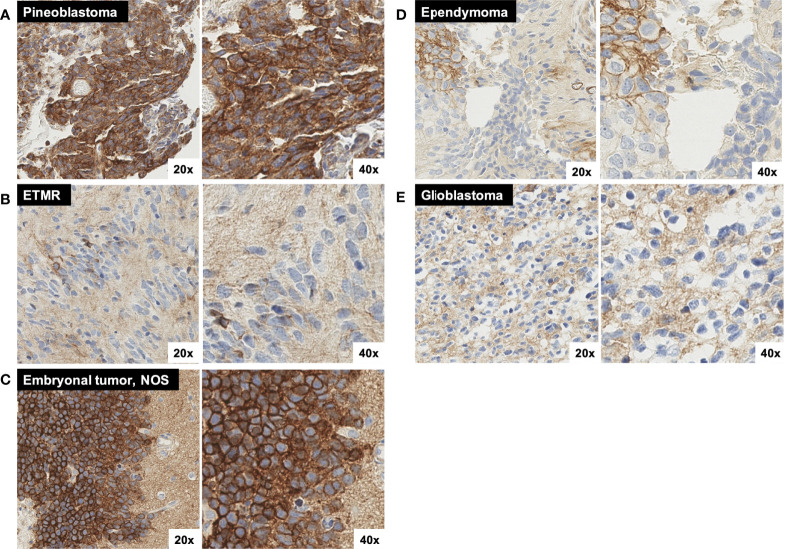
Additional representative SST2A IHC images. **(A)** Pineoblastoma sample with moderate to strong SST2A staining in 10-50% of tumor cells. **(B)** ETMR sample with very small (<<10%) areas of light and/or incomplete membranous positivity, with otherwise absent tumoral staining throughout. Background granular and/or cytoplasmic staining is demonstrated. **(C)** Embryonal tumor, NOS with strong SST2A staining intensity in nearly 100% of tumor cells. Tumor cells (left) are shown in proximity to background cortical tissue (right), which demonstrates non-specific (non-membranous) staining. **(D)** Ependymoma (grade II) sample with very small (<10%), focal areas of membranous positivity (on upper left), but otherwise absent tumoral staining. Background granular and/or cytoplasmic staining is demonstrated. **(E)** Glioblastoma sample with minimal to absent tumoral membranous staining, yet diffuse background staining of non-neoplastic tissue. All images are shown at 20x and 40x magnification.

All four (100%) pineoblastoma tumors had positive SST2A IHC scores, with at least moderate staining intensity in each specimen (**Figure 2**). The two tumors with higher membranous SST2A expression [both with scores of 5 (**Figure 5A**)] had diffuse leptomeningeal metastases at diagnosis; in one of these specimens, SST2A IHC positivity appeared to correlate with focal papillary morphology. The remaining two tumors (with scores of 2 and 3, both with localized pineal disease) exhibited small (<10%), focal areas of moderate to strong SST2A expression (**Figure 1J**). All four patients are alive without evidence of recurrence.

#### ATRT and ETMR

Negative SST2A IHC scores (0–1) were observed in all six (100%) cases of atypical teratoid/rhabdoid tumor (ATRT) and embryonal tumor with multilayered rosettes (ETMR), irrespective of sex, age, metastatic disease, or likelihood of relapse (**Figure 2**). The three ATRT tumors exhibited largely absent membranous SST2A expression, with scores of 0 in two samples (**Figure 1D**) and at most 1 in the remaining specimen. Minimal SST2A expression was similarly demonstrated in the three ETMR tumors, which all received scores of 1 for very small (<10%) areas of light and/or incomplete membranous positivity, with otherwise absent staining throughout (**Figure 5B**). Morphological rosette structures lacked membranous SST2A IHC positivity.

#### Other embryonal tumors

Among seven additional embryonal tumors not classified in the above histologic categories (i.e., most formerly diagnosed as CNS primitive neuro-ectodermal tumor [PNET]), there was heterogeneous membranous SST2A expression, with positive IHC scores in two specimens (29%) (**Figure 2**). Notably, one tumor, characterized histologically as malignant embryonal tumor, not otherwise specified (NOS), with especially primitive morphology, received one of the highest IHC scores of the entire cohort, with nearly 100% membranous immunoreactivity of strong intensity (**Figure 5C**); this patient presented in adolescence with extensive intracranial and spinal metastases and remains recurrence-free approximately one year post-completion of craniospinal irradiation and adjuvant chemotherapy. The other embryonal tumor with SST2A IHC positivity (score of 2, primary supratentorial location) exhibited focal areas of moderate staining intensity (**Figure 1E**). Minimal to absent membranous SST2A expression was observed in the remaining five embryonal tumor specimens). Neither genetic sequencing nor methylation testing was available on these tumors, limiting further molecular characterization.

### Meningiomas

Membranous SST2A expression was consistently identified in meningiomas (**Figure 2**), with positive SST2A IHC scores in 14 of 15 (93%) tumors analyzed (**Figures 1C, F, L**). Scores were variable, ranging from 2 to 11, with no significant correlation with histologic grade (grade I: 5.2 ± 3.7 [n=6]; grade II: 5.6 ± 3.4 [n=8], p=0.81 [the one grade III meningioma received a score of 9]). There was no association between SST2A IHC score and patient age at diagnosis, gender, likelihood of progressive disease, or prior treatment exposure (p>0.05 for all). Intratumoral heterogeneity of membranous SST2A expression was frequently observed, with focal areas of positivity in several cases. In at least one tumor (grade II), SST2A staining correlated with morphology, present on most meningioma cells with classic appearance and absent in most spindle-shaped, sarcomatous cells, but this was not universally seen.

### Ependymomas

Minimal to absent membranous SST2A expression was demonstrated in the 27 ependymoma samples evaluated (**Figure 2**), with negative SST2A scores in 25 (93%) tumors (**Figure 1A**). The remaining two ependymomas (grade II) had scores of 2 and 3, respectively, with very small (<10%), focal areas of membranous (**Figure 5D**). Largely absent SST2A expression was consistently observed across ependymomas of different histologic grades (grades I-II: 0.2 ± 0.7 [n=18]; grade III/anaplastic: 0.3 ± 0.7 [n=9]; p=0.57) and primary tumor locations (posterior fossa: 0.2 ± 0.5 [n=19]; supratentorial: 0.5 ± 1.2 [n=6]; p=0.32). Six tumors had molecular profiling (posterior fossa group A [n=3], posterior fossa group B [n=2], supratentorial *ZFTA-RELA* fusion-positive [n=1]), all with SST2A IHC scores ≤2.

### High-grade gliomas

Membranous SST2A expression was minimal to absent in the 23 pediatric high-grade gliomas analyzed (**Figures 1B, I**, **Figure 2**), which included the following specific histologic diagnoses: (a) anaplastic astrocytoma, anaplastic pleomorphic xanthoastrocytoma, and anaplastic oligodendroglioma (n=5), (b) high-grade glioneuronal tumor (n=2), (c) glioblastoma (n=9), (e) non-pontine diffuse midline glioma, H3 K27M-mutant (n=3), and (f) DIPG (n=4; diffuse midline glioma, H3 K27M-mutant [n=3] and anaplastic astrocytoma [n=1]). Twenty-one (91%) tumors had negative SST2A IHC scores; the remaining two high-grade glioma samples received scores of 2 (both glioblastoma, one of which was *IDH1*-mutant), with interpretation limited somewhat by specimen quality and diffuse non-tumoral, non-membranous background staining (see below) in both cases. Negative SST2A scores were consistently observed across the aforementioned five high-grade glioma diagnosis subgroups, without differences by specific histology.

### Paired diagnostic and recurrent tumor samples

**Figure 6:**
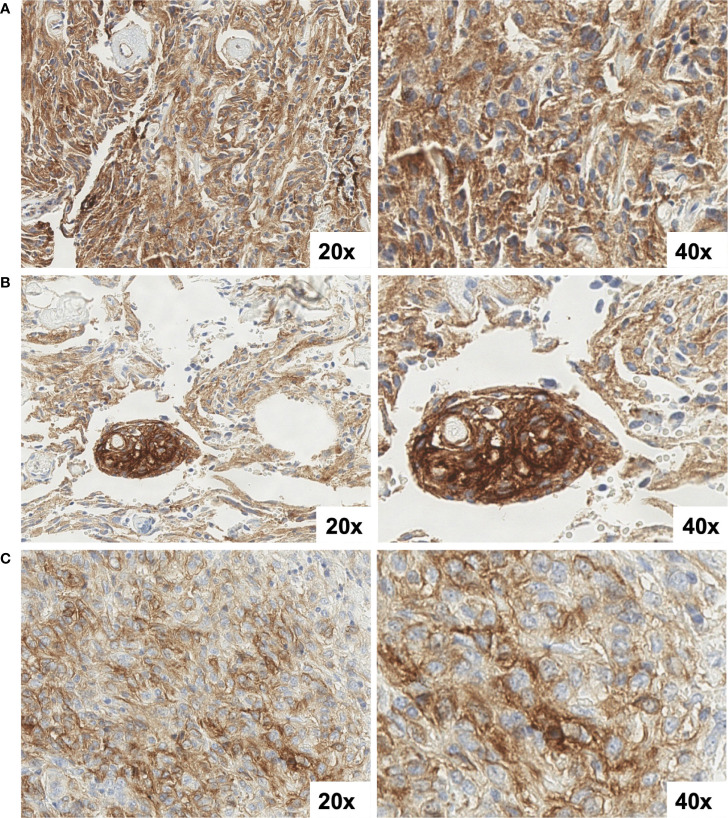
SST2A IHC images of a multiply recurrent atypical meningioma from three time-points: **(A)** initial diagnosis, prior to treatment, **(B)** post-radiation, five years after diagnosis, and **(C)** following re-irradiation two years later **(C)**. All specimens demonstrated 10-20% tumor cell immunoreactivity and intratumoral heterogeneous staining intensity, ranging from light to strong. Non-specific background staining is also observed. All images are shown at 20x and 40x magnification.

Four patients had paired diagnostic and recurrent tumor specimens analyzed, enabling preliminary assessment of temporal heterogeneity in SST2A expression profiles. SST2A IHC scores were largely conserved over time within this small subset. One patient with a multiply progressive atypical meningioma had three tumor specimens evaluated, all with similar membranous SST2A expression: 10-20% tumor cell immunoreactivity and intratumoral heterogeneous staining intensity (light to strong) was demonstrated in each of the diagnostic and two recurrent specimens (latter following radiation and reirradiation, respectively) (**Figure 6**). The remaining paired specimens all exhibited conserved absence of SST2A expression. Specifically, two patients with recurrent ependymomas (grade II and grade III/anaplastic) had specimens submitted from diagnosis and post-radiation local relapses, all with SST2A IHC scores of zero. One patient with an H3 K27M-mutant diffuse midline glioma had tumor specimens evaluated from diagnosis (pre-treatment biopsy of spinal lesion) and metastatic progression (biopsy of extraneural osseous metastasis), both entirely lacking membranous SST2A expression.

### Association between membranous SST2A expression and survival:

Preliminary analyses evaluating association between SST2A IHC score and outcomes (event-free survival and overall survival) were performed, recognizing interpretation is limited by the relatively low rates of recurrence/progression and death in the cohort as well as by heterogeneity in histologic diagnoses. When assessed in univariate analyses across the entire cohort (all histopathologic diagnoses), SST2A IHC score (analyzed as a continuous variable) was associated with improved event-free survival (HR=0.85 [95% CI: 0.76-0.96]; p=0.009) and overall survival (HR=0.64 [0.42-0.97]; p=0.034). However, in multivariable analyses adjusting for histopathologic diagnosis (i.e., classification as embryonal tumor, meningioma, ependymoma, or high-grade glioma), SST2A IHC score was no longer predictive of event-free survival (HR=0.87 [0.74-1.01], p=0.07), but remained associated with overall survival (HR=0.33 [0.12-0.95]; p=0.039).

### Inter-rater reliability

Among the subset of 50 tumors reviewed (via digital slide upload) in blinded fashion by a second pathologist, there was moderate agreement in ordinal score measurements, with a weighted kappa of 0.54 (p<0.0001), and strong positive correlation in score absolute values (Spearman’s Rho=0.81, p<0.0001). Subsequent retrospective review of discordant cases by the two pathologists revealed that most discrepancies occurred in cases with poor, compromised tumor tissue quality and/or diffusely positive background, non-membranous staining which potentially limited visualization of the tumor cell surface.

## Discussion

Potential incorporation of somatostatin receptor-targeted therapy in the treatment of children, adolescents, and young adults with refractory CNS tumors requires an understanding of the prevalence and key correlates of SST2A expression across these aggressive diseases. To our knowledge, this study represents one of the first detailed immunohistochemical assessments of membranous SST2A expression within a representative cohort of pediatric high-risk CNS tumors utilizing previously validated, functionally-relevant IHC scoring methodology. Whereas SST2A was largely absent from the tumoral cell surface of pediatric high-grade gliomas and ependymomas, high membranous SST2A expression was demonstrated in medulloblastoma, meningioma, and some rarer embryonal tumors with important diagnostic, biologic, and therapeutic implications.

Medulloblastomas in our cohort consistently expressed tumoral cell surface SST2A, in accord with previous reports describing SST2A expression within this histopathologic diagnosis when evaluated through a combination of different assays, including IHC, mRNA levels, somatostatin receptor autoradiography, somatostatin receptor scintigraphy, and/or SST2A-radiolabeled nuclear imaging (e.g., DOTATATE PET) (13–16, 20, 21, 26–31). Our findings confirm and expand upon this earlier work by illustrating a high prevalence of membranous (i.e., functional and targetable) SST2A protein expression among medulloblastoma cases assessed by strict immunohistochemical measures. More than 90% of the 38 analyzed medulloblastoma specimens had positive SST2A IHC scores, all with moderate-strong staining intensity and most exhibiting >50% tumor cell immunoreactivity.

Although nearly all medulloblastoma tumors expressed membranous SST2A to some extent, differences in expression were detected across histopathologic and molecular subgroups. The highest SST2A IHC scores were observed in large cell/anaplastic tumors (albeit the smallest sample size), followed by classic histology, and lowest in the nodular/desmoplastic variant. Correspondingly and in agreement with the findings of Remke et al. (29), non-SHH medulloblastoma tumors had significantly higher membranous SST2A expression than SHH tumors, with a trend toward greater SST2A IHC scores in cases further classified by methylation testing as group 3. Additionally, SST2A IHC positivity correlated with morphology in some medulloblastoma specimens, most notable when assessed across the intratumoral spectrum of differentiation within nodular/desmoplastic histology. In several nodular/desmoplastic tumors (including two infant SHH cases with higher SST2A IHC scores), more immature cells highly expressed membranous SST2A, whereas more differentiated cells comprising the nodules lacked membranous SST2A positivity (yet exhibited cytoplasmic granularity). These findings provide support for a proposed association between SST2A overexpression and genomically-defined dedifferentiated, proneural, and/or primitive neuronal precursor lineage from studies in adult CNS tumors (37). However, an almost inverse SST2A IHC staining pattern was occasionally noted (including in one adolescent SHH tumor which received a low IHC score) with absent SST2A expression in more primitive cells, yet positivity (however still usually cytoplasmic, not membranous) in the nodule’s maturing cells. This variation of SST2A expression both within SHH (known to comprise many subtypes [e.g., SHH-γ, SHH-α]) and across medulloblastoma subgroups likely reflects the biological intra- and inter-tumoral heterogeneity of this disease (59, 60), for which the emerging oncogenic role of somatostatin receptor pathways warrants continued research.

There is a critical need to develop novel therapies for children and young adults with relapsed medulloblastoma, who currently have limited treatment options, no standard salvage regimen, and an especially poor prognosis, with overall survival <15% (3, 5, 6). Promising results of somatostatin receptor-targeted therapy (both somatostatin analogues like octreotide as well as SST2A peptide receptor radionuclide treatment) have been observed in small series of recurrent medulloblastoma cases, with sustained radiographic and clinical responses in tumors refractory to conventional radiation and chemotherapy (40–42). Moreover, positive correlations between extent of membranous SST2A expression, evaluated using similar immunoreactive IHC scoring as implemented here, and response to somatostatin receptor-targeted therapy were detected in gastrointestinal neuroendocrine tumors and pituitary adenomas (51, 58, 61, 62). Improved response rates, progression-free survival, and overall survival following such treatment were demonstrated in tumors with SST2A IHC scores of at least 5-6 (51, 61), which corresponds to >75% of the medulloblastoma cases in our cohort, further supporting investigation of somatostatin receptor-targeted therapy such as ^177^Lu-DOTATATE in these patients. Development of an early phase clinical trial of ^177^Lu-DOTATATE in children and young adults with refractory SST2A-expressing high-grade CNS tumors, including medulloblastoma, is currently underway, using the immunohistochemical evaluation of membranous SST2A expression described here for eligibility screening (NCT05278208). If somatostatin receptor-targeted therapy proves effective in medulloblastoma and other SST2A-expressing CNS tumors, this could eventually be incorporated into upfront treatment backbones for these aggressive diseases, potentially presenting a modality to deliver targeted, localized radiation in younger patients with high-risk tumors. Importantly, prevalent membranous SST2A expression was observed in aforementioned medulloblastoma tumors with known poor prognostic molecular, histopathologic, and clinical features (group 3, large cell/anaplastic histology, metastatic disease) (63, 64), representing a possible therapeutic target in the upfront setting for these more challenging subgroups.

Heterogeneous membranous SST2A expression was identified across other pediatric embryonal tumors. Earlier reports described mixed results regarding SST2A expression by IHC or mRNA in small series of supratentorial CNS-PNETs, with SST2A positivity noted in some studies (17, 28, 31), but absent expression in others (29). Our findings expand upon this previous work, demonstrating varied membranous SST2A expression in non-medulloblastoma embryonal tumors—present in pineoblastoma, absent in ATRT and ETMR, wide-ranging in remaining cases. Despite shared histopathologic features, the observed heterogeneity in SST2A expression among these rarer pediatric embryonal tumors likely parallels their divergent molecular landscapes and distinct DNA methylomes (65–68). Detailed genetic sequencing or methylation array were not available on these tumors, precluding molecular characterization. Further exploration in a larger cohort with comprehensive genomic profiling is necessary, but these results suggest a potential role for somatostatin receptor-targeted therapy in certain embryonal tumors, including pineoblastoma.

Membranous SST2A expression was prevalent in pediatric meningiomas in our cohort, with positive IHC scores in nearly all cases. These findings corroborate previous reports of SST2A overexpression in most meningiomas with corresponding uptake on somatostatin-receptor radiolabeled nuclear imaging (25, 32, 33). Whereas this earlier work largely focused on meningiomas in adult patients, we assessed SST2A expression in meningiomas diagnosed during childhood, adolescence, or young adulthood (median age at diagnosis: 14 years [range: 6-29]), with similar IHC positivity as older counterparts. Although most pediatric meningiomas expressed SST2A to some extent, both intra- and inter-tumoral heterogeneity in expression was observed. Potential correlations between immunohistochemical SST2A expression and histologic grade, microvessel density, and/or morphologic features have been suggested in adult meningiomas (25, 69, 70), yet not consistently shown. We did not identify significant associations between meningioma SST2A IHC scores and histopathologic or clinical characteristics, albeit possibly limited by the small sample size and thus deserving continued investigation. Nonetheless, pediatric and young adult patients with recurrent meningiomas face poor outcomes with limited effective treatments, especially in cases where surgery and conventional radiation are not feasible or confer excessive toxicity (25, 71, 72). Somatostatin receptor-targeted therapy represents a promising consideration for refractory and/or unresectable pediatric meningiomas, given frequently detected membranous SST2A expression in these tumors as well as emerging reports of response or prolonged disease stabilization in treated adult patients (22–25).

Minimal to absent membranous SST2A expression was consistently demonstrated in all pediatric ependymomas and high-grade gliomas in our cohort, irrespective of histology, tumor location, or patient clinical features. Although prior reports describe the presence of SST2A within some pediatric ependymomas and high-grade gliomas (17, 27, 34, 39), positive findings were largely limited to cytoplasmic IHC staining and/or mRNA expression, which exhibited poor correlation with membranous immunolabeling and functional protein levels, likely due to post-translational modification (39, 47, 48). Our results confirm the general paucity of targetable, tumoral cell-surface SST2A in pediatric ependymomas and high-grade gliomas seen in earlier studies (17, 27, 29, 38, 39), evaluated here through utilization of stringent IHC measures, a more specific, monoclonal anti-SST2A antibody, and a larger sample size. Whereas membranous SST2A expression was lacking, many pediatric high-grade glioma specimens in our cohort [especially those classified histologically as glioblastoma or diffuse midline glioma, H3 K27M-mutant (**Figure 5E**)] as well as some ependymomas (**Figure 5D**) exhibited non-specific, background staining—suspected to represent normal glial processes in close proximity to malignant cells in these highly infiltrative tumors and/or endothelial and inflammatory cells in the setting of prominent vascular proliferation or necrosis, in accord with Cervera et al. (27).

Additionally, within a small group of patients with paired diagnostic and recurrent tumor specimens analyzed, SST2A expression profiles were conserved over time, including after treatment. Although interpretation is limited by the sample size and absent expression in most paired specimens (ependymomas and high-grade gliomas), these results support lack of temporal heterogeneity in tumoral SST2A, yet continued research in a larger cohort of recurrent tumors will be necessary.

The favorable prognostic impact of SST2A overexpression in gastrointestinal neuroendocrine tumors, neuroblastoma, and adult anaplastic oligodendrogliomas has been demonstrated (36, 37, 51–55), but little outcome data has been reported thus far within pediatric neuro-oncology. Remke et al. showed improved overall survival in medulloblastoma cases with high (>50% immunoreactivity) SST2A levels (significant correlation in their institutional cohort, trend in their larger validation cohort) (29). We observed a correlation between higher SST2A IHC scores with increased event-free survival and overall survival in univariate analyses when evaluated across the entire cohort, though in multivariable analyses adjusting for histolopathology, only the association with overall survival remained significant. Caution must be applied when drawing conclusions due to the relatively low number of patients with recurrent/progressive disease or death as well as significant heterogeneity of assesed histologic diagnoses and corresponding outcomes. However, these preliminary findings suggest potential prognostic significance of membranous SST2A expression within some high-risk pediatric CNS tumors that demands further investigation and corroboration in larger-scale studies.

Finally, in a subset of tumors with SST2A IHC interpreted by two pathologists (blinded to one another), moderate inter-reviewer reliability, with strong correlation in score absolute values, was demonstrated. Discordant impressions of membranous SST2A positivity were limited to cases of poor, compromised tumor tissue quality and/or diffusely positive background, non-membranous staining, indicating potential for technical and biological factors to impede interpretation; adjudication by consensus between two reviewers is likely necessary in these rare, but challenging cases. This further highlights the importance of IHC-based scoring being performed by a neuropathologist highly familiar with SST2A staining patterns, especially when results may have therapeutic implications. This is the case for the aforementioned clinical trial investigating ^177^Lu-DOTATATE in high-risk pediatric CNS tumors (NCT05278208), which mandates central pathology review as part of eligibility screening. Additionally, SST2A IHC scoring excludes inevitable non-specific background staining commonly encountered in IHC methodology in general (73), as well as cytoplasmic positivity seen in diseased or healthy brain tissue, likely corresponding to endothelium, inflammatory cells, and/or glial processes, as described herein and in other studies (27, 74, 75). This non-membranous (and thus non-targetable) staining should not confer increased toxicity risk with ^177^Lu-DOTATATE, but given the potential to confound IHC impressions, efforts should be taken to select an anti-SST2A antibody with superior binding affinity and minimal cross-reactivity with other antigens. The commercially available rat monoclonal anti-SST2A, UMB-1, was used in the present study because it has demonstrated more distinct membranous staining and less diffuse background staining compared to alternative agents (49, 50, 76).

This study expands our understanding of the prevalence, correlates, and therapeutic implications of membranous SST2A expression across high-risk pediatric CNS tumors. Medulloblastoma (especially non-SHH subgroups), meningioma, and some rarer embryonal tumors highly expressed SST2A, suggesting a potential role for somatostatin receptor-targeted therapy such as ^177^Lu-DOTATATE in these aggressive diseases. Pediatric ependymomas, high-grade gliomas, ATRT, and ETMR consistently lacked membranous SST2A expression. SST2A variation within and across these histopathologic diagnoses provides valuable insight into their underlying biological and molecular heterogeneity. Taken together, these findings support utilization of membranous SST2A as a diagnostic tool, therapeutic target, and potential biomarker in some high-risk pediatric CNS tumors, which will be essential to explore in future research.

## Data availability statement

The original contributions presented in the study are included in the article/supplementary material. Further inquiries can be directed to the corresponding author.

## Ethics statement

The studies involving human participants were reviewed and approved by Cincinnati Children’s Hospital Medical Center IRB. Written informed consent to participate in this study was provided by the participants’ legal guardian/next of kin.

## Author contributions

All authors contributed to study design. ML and SS analyzed all SST2A IHC cases. CF analyzed a subset of cases. ML abstracted clinical data and performed analyses, with statistical guidance from JS. All authors participated in interpreting results, writing the manuscript, and revising the manuscript critically for important intellectual content All authors contributed to the article and approved the submitted version.

## Funding

The Pray Hope Believe Foundation (PI: Salloum).

## Acknowledgments

We thank all the patients and families who contributed tumor tissue for their generous donation to this research. We also thank The Pray Hope Believe Foundation for their support of this study. We acknowledge Betsy DiPasquale and Christopher Woods in the Department of Pathology at CCHMC for their technical assistance.

## Conflict of interest

The authors declare that the research was conducted in the absence of any commercial or financial relationships that could be construed as a potential conflict of interest.

## Publisher’s note

All claims expressed in this article are solely those of the authors and do not necessarily represent those of their affiliated organizations, or those of the publisher, the editors and the reviewers. Any product that may be evaluated in this article, or claim that may be made by its manufacturer, is not guaranteed or endorsed by the publisher.
